# Presence of *Helicobacter pylori* and *Campylobacter ureolyticus* in the oral cavity of a Northern Thailand population that experiences stomach pain

**DOI:** 10.1080/20002297.2018.1527655

**Published:** 2018-10-17

**Authors:** Amina Basic, Hanna Enerbäck, Sara Waldenström, Emma Östgärd, Narong Suksuart, Gunnar Dahlen

**Affiliations:** a Department of Oral Microbiology and Immunology, Institute of Odontology, Sahlgrenska Academy, University of Gothenburg, Gothenburg, Sweden; b Princess Mother Medical Voluntary Foundation, Bangkok, Thailand

**Keywords:** Stomach pain, Karen Hill tribe, caries, periodontal disease, oral microbiota, *Helicobacter pylori*, *Campylobacter ureolyticus*

## Abstract

**Objective**: To investigate oral diseases and microbiological conditions, such as the presence of ureolytic bacteria in dental plaque, in relation to experience of stomach pain in a remote adult Asian population.

**Methods**: Ninety-three adults, 40–60-years old, from the Karen Hill tribe in Northern Thailand with no regular access to dental care were examined. Clinical registrations were performed and interproximal gingival plaque samples were collected and analyzed with the checkerboard (CKB) method for the presence of 14 oral bacterial species.

**Results**: A number of 61 subjects reported daily stomach pain while 32 subjects had no symptoms from the stomach. The subjects with stomach pain had fewer remaining teeth (*p* < 0.05), higher caries experience (*p* < 0.05) and less BoP (*p* < 0.01). Most of the bacterial species were clustered statistically in three factors in a factor analysis, which together explained 65% of the microbiological variance. Factor 1, explaining 43.0% of the variance, was statistically associated with stomach pain (*p* < 0.001).

**Conclusions**: The interproximal plaque/biofilm in adults of the study population showed a common presence of two gastrointestinal pathogens *H. pylori* and *C. ureolyticus. *The study also indicates for the first time a potential association between *C. ureolyticus* and stomach pain.

## Introduction

Indigestion or dyspepsia is a common condition with persistent pain or discomfort localized to the upper part of the stomach [1]. It implicates several different medical conditions such as gastritis and peptic ulcer. Approximately, half of the world population is colonized with *Helicobacter pylori* in the stomach [2]. This bacterial species seems to be more prevalent in developing countries where up to 80% of the children harbor the bacterium in the stomach [3,4]. Infection with *H. pylori* is treated with antibiotics but reinfection after treatment is common. Previous studies have detected *H. pylori* in the oral cavity [5,6] and examined the presence of *H. pylori* in saliva or dental biofilm in association with oral hygiene [6]. The presence of *H. pylori* in saliva and dental biofilm has been reported to be between 0 and100%, depending on the population studied and the method used for detection of the bacteria [6]. It has been suggested that the oral cavity can function as a reservoir for *H. pylori* and thereby contribute to reinfection of the stomach. Conversely, other reports claim that the oral cavity is a transient passage for the bacterium [6].

The main characteristic of *H. pylori* is its highly ureolytic capacity, by which it can convert urea into ammonia and carbon dioxide and thereby increase the pH of its surroundings [7]. This characteristic makes it possible for the bacteria to withstand the acidic environment in the stomach. This could also be of significance for the bacteria to survive in the dental plaque, which frequently is acidogenic after exposure to sugars from the diet. No associations have yet been reported between the presence of *H. pylori* and caries prevalence. *H. pylori* may however, when present in the subgingival plaque, contribute to the more alkaline environment found in the gingival pocket and thus be associated with periodontal disease [6]. Periodontal disease is common and affects most middle-aged individuals worldwide [8]. The periodontal pockets may harbor complex collections of several hundreds of different microorganisms in the anaerobic environment found at the diseased periodontal sites [9]. The periodontal pocket has been suggested to be a potential natural reservoir for *H. pylori* [10,11]. Similarly, poor oral hygiene may predispose or favor the presence of *H. pylori* in individuals with abundant dental plaque and calculus.

*Campylobacter ureolyticus* is another highly ureolytic bacterial species that is suggested to be a gastrointestinal pathogen [12,13]. It is occasionally isolated from the dental plaque of periodontitis patients [14]. This bacterial species, previously known as *Bacteroides ureolyticus*, is a strict anaerobic, Gram-negative rod with corroding characteristics when grown on blood agar plates [15]. However, little is known about its prevalence in dental plaque, its potential presence in the stomach, and its possible association with stomach pain.

*H. pylori* infection is common in Southeast Asia [2,4], as is periodontal disease [16,17]. Caries prevalence is, however, low in populations of remote areas [18,19]. In previous studies, conducted on the Karen Hill population of Northern Thailand, it was reported that *H. pylori* and *C. ureolyticus* were present in subgingival plaque samples, and its presence was associated to the pH and the ureolytic capacity of the plaque [18,20] in the adult population. It is therefore reasonable to assume that the presence of these ureolytic bacteria in the oral cavity would be more frequent in subjects experiencing stomach pain. We hypothesized that a higher prevalence of *H. pylori* and *C. ureolyticus* is found in the dental plaque microbiota of adults with stomach pain compared to individuals with no stomach pain.

The aim of this study was to investigate the microbiological (using DNA–DNA hybridization) and physiological (pH and ureolytic activity) patterns in the interproximal plaque/biofilm of adults with or without caries experience and periodontal disease in relation to stomach pain. Special attention was paid to the presence of the strong ureolytic species *H. pylori* and *C. ureolyticus*.

## Material and methods

### Study subjects

The examination and collection of samples in this study was carried out at 2 different occasions in 11 villages that belong to the Karen Hill Tribe in the Omgoi district of Northern Thailand. The study was conducted in collaboration with a mobile team of dentists and medical doctors from Princess Mother Medical Voluntary Foundation (Bangkok, Thailand) that also ethically approved the study. Inclusion criteria were subjects between 40 and 60 years of age. The participants were consecutively included in the study. A total of 93 individuals (50 at the first occasion and 43 at the second occasion) were informed of the study and voluntarily consented to participate. The subjects were interviewed for their stomach problems with an interpreter, who spoke the local language. Although the stomach pain was self-reported, a medical doctor confirmed that gastritis or peptic ulcer was likely. The subjects, who reported daily stomach pain, were included in the stomach pain group and subjects reporting no or infrequent stomach pain were included in the control group. The individuals were interviewed on their oral hygiene habits, tobacco use, betel chewing habits, and intake of sugar.

### Clinical examination

The clinical examination was performed in natural light with the help of a mouth mirror and a dental and periodontal probe prior to the examination and treatment performed by the mobile dental team. The examination included registration of dental status (number of remaining teeth), plaque index (PI), decayed, filled teeth (DFT), bleeding on probing (BoP), probing pocket depth (PPD), clinical attachment level (CAL), and calculus according to methods and criteria used in previous studies [17–20]. The participants were divided into three groups according to their periodontal status; gingivitis (with PPD ≤4 mm), mild periodontitis (at least one pocket with PPD >4 mm but <7 mm), and severe periodontitis (at least one pocket with PPD ≥7 mm or suspected loss of teeth due to periodontitis).

Calculus was scored as 0, 1, or 2 in six areas of the mouth, where 0 = free of calculus, 1 = calculus around the gingival margin, and 2 = significant quantity of calculus. At the level of the individual, calculus was scored as 0 = up to grade 1 in one sextant, 1 = grade 1 in at least two sextants or grade 2 in one sextant, 2 = grade 2 in at least two sextants [21].

The urease activity of the dental biofilm was measured by the use of a modified rapid urease test [18,22,23] in interproximal plaque samples from 4 interproximal sites (mesial aspect of 11, 26, 36, and 41). The urea broth used consisted of urea (10%) in distilled water and 0.02 g/L of phenol red; the pH was adjusted to 6.8. The broth was used in 100 µL volumes to which a loopful (1 µL Inoculation loop, Sarstedt, Nümbrecht, Germany) of dental plaque was added. The color production of the assay was evaluated after 2 h in a temperature of 20–25°C. The color change was divided according to a 4-graded scale [23], where 0 corresponded to no reaction (colorless), 1 corresponded to a pink reaction, 2 to a red reaction, and grade 3 corresponded to a deep purple reaction. Author GD performed the color readings.

The pH of the dental biofilm was measured at baseline at 4 interproximal sites (the mesial aspect of 16, 21, 31, and 46) with the use of the pH-strip method [24]. pH registrations where performed with pH indicator strips cut into an arrow shape to facilitate interproximal placement. When teeth at the interproximal sites were missing, the registration was conducted at the nearest approximal site.

Microbiological plaque samples were collected at four interproximal sites (the distal aspect of 11, 26, 31, 46) with a curette. The samples were transferred to Eppendorf tubes containing 100 µL of TE buffer (0.5 mm Tris-EDTA) and to which 100 µL 0.1 M NaOH was added. The samples were analyzed at the Oral Microbiological Diagnostic Service Laboratory, Department of Oral Microbiology, Institute of Odontology at Gothenburg University, Sweden.

### Microbiological analyses

Whole-genomic DNA probes were prepared for common dental plaque bacteria and bacteria with known ureolytic activity [18,23]. The species were *Actinomyces oris, Campylobacter gracilis, Campylobacter rectus, C. ureolyticus, Filifactor alocis, Fusobacterium nucleatum, H. pylori, Haemophilus parainfluenzae, Lactobacillus fermentum, Prevotella intermedia, Prevotella tannerae, Streptococcus mutans, Streptococcus salivarius*, and *Streptococcus sanguinis* [18,20]. DNA was extracted with mutanolysin and lysozyme as previously described [25] and the quality of the DNA was evaluated with ultraviolet (UV) spectrum using a GeneQuant spectrophotometer (Pharmacia Biotech, Uppsala, Sweden). DNA probes (1 µg) were labeled with deoxygenin using the DIG High Prime kit according to the manufacturer’s instructions (Roche Diagnostics, Mannheim, Germany). The analyses were conducted using checkerboard (CKB) DNA–DNA hybridization methodology specifically adapted for Gram-positive microorganisms according to Wall-Manning et al. [26]. All probes were cross-tested against all bacterial species included in the panel to check cross-hybridization. The plaque samples were boiled for 5 min and neutralized with 800 µL 5 M ammonium acetate. Aliquots of 150 µL of samples were transferred onto nylon membranes (Minislot device, Immunetics, Cambridge, MA) and fixed by UV light (UV Stratalinker 1800, Stratagene, La Jolla, CA). After 2 h of pre-hybridization at 42°C, the DNA probes (1–10 ng) were allowed to hybridize overnight in lanes vertical to the plaque samples using a Miniblotter device (Immunetics) at 42°C. After a series of stringency washes at 70°C, hybrids were detected using phosphatase-conjugated anti-digoxigenin antibodies and the signals were visualized with a chemiluminescent substrate (CDP Star, Roche Diagnostics). The number of bacteria in the samples was compared to standard samples containing 10^6^ and 10^5^ cells of each species [27]. The readings were performed with the visual score method (0–5) and using the percent method (based on BLU-signals intensities expressed as a fraction of the signal intensity of the high standard) according to Dahlén et al. [25] as control.

### Statistical analysis

All statistical analyses were performed in IBM SPSS Statistics Software (Version 21, Chicago, IL). The identified bacterial counts were scored as 0 (no reaction), score 1 (<10^5^ cells), score 2 (10^5^ cells), score 3 (>10^5^ cells), score 4 (10^6^ cells), and score 5 (>10^6^ cells) for overview but recoded into log counts before a factor analysis was performed on site level with Varimax rotation. The relationship between urease activity (yes/no), *H. pylori, C. ureolyticus*, pH, and PPD was explored with Chi-square test for independence (with Yates Continuity Correction). Stomach pain was studied with logistic regression analyses where subject variables were included in the model. The same test was also used to investigate urease activity and the clinical findings for each site. A *p*-value of less than 0.05 was considered statistically significant.

## Results

### Clinical findings

Ninety-three subjects chose to participate. Sixty-one of them (66%) reported to have daily stomach pain. The two groups (stomach pain/no stomach pain) were fairly similar in gender distribution, age, use of betel and smoking, oral hygiene habits, and sugar intake (Table 1). Remaining teeth were significantly lower (*p* < 0.05) and caries frequency (DFT) significantly higher in the stomach pain group compared to those without experience of stomach pain. Furthermore, their clinical oral status was similar with regard to PI, CAL, and Calculus. BoP was, however, significantly lower for the stomach pain group compared with the group experiencing no stomach pain (*p* < 0.01). Periodontal disease (mild and advanced) was more prevalent in the stomach pain group (PPD >4 mm, 67 vs. 50%) although not statistically significantly different. Interproximal pH showed a large variation but was generally slightly alkaline (>7.0) for all individuals of both groups. This was most expressed at the sites 31 and 16 (data not shown) and in some cases, a pH >8.0 was noted. Urease activity was found in a broad range at all sites in both groups and did not correlate with any site parameter or bacteria (data not shown).

**Table 1.** Characteristics of the 93 subjects examined, divided into 2 groups: those experiencing frequent stomach pain and those never or very rarely experiencing stomach pain.

Variable		Stomach pain		
		Yes (*n* = 61)	No (*n* = 32)	
Women (%)		37 (61)	18 (56)	
Age (mean ± SD^a^)		51.0 ± 6.5	50.6 ± 7.1	
Betel chewing (%)		36 (59)	17 (53)	ns
Tobacco smoking (%)		32 (52)	19 (59)	ns
Both betel and smoking (%)		14 (23)	12 (38)	ns
Oral hygiene habits (%)^b^	Never	24 (41)	11 (33)	ns
	Sometimes	17 (29)	9 (28)	
	Every day	17 (29)	12 (38)	
Sugar intake (%)	Never	11 (18)	3 (9)	ns
	Once a week	34 (56)	19 (59)	
	Every day	16 (26)	10 (30)	
Remaining teeth (mean ± SD)		25.6 ± 4.0	26.9 ± 1.7	*
DFT^c^ (mean ± SD)		4.9 ± 8.1	2.3 ± 3.4	*
PI^d^ (mean ± SD)		87.8 ± 14.8	91.6 ± 8.1	ns
BoP^e^ (mean ± SD)		48.9 ± 34.5	74.8 ± 32.4	**
Calculus median (range)^f^		4 (0–8)	4 (1–8)	ns
Subjects with PPD (%)^g^	>4 < 7 mm	25 (41)	10 (30)	ns
	≥7 mm	16 (26)	6 (19)	
Subjects with CAL (%)^h^	>3 < 7 mm	29 (48)	14 (44)	ns
	≥7 mm	17 (28)	8 (25)	
pH mean (range)^i^		7.2 (6.5–8.4)	7.2 (6.5–8.2)	ns
Urease test score (range)^j^		0 (0–1)	0.5 (0–2)	ns

*p < 0.05.

** *p* < 0.01.

^a^Standard deviation.

^b^*n* = 90. Missing data on three individuals.

^c^Decayed filled teeth.

^d^Plaque index.

^e^Bleeding on probing.

^f^*n* = 74. Missing data on 19 individuals.

^g^At least one pocket with PPD (probing pocket depth) >4 < 7 mm or ≥7 mm.

^h^At least one pocket with CAL (clinical attachment level) >3 < 7 mm or ≥7 mm.

^i^Mean calculated from mean of four measuring sites per subject, *n* = 92.

^j^Median calculated from median of four measuring sites per subject, *n* = 66.

### Microbiological findings

**Figure 1.**
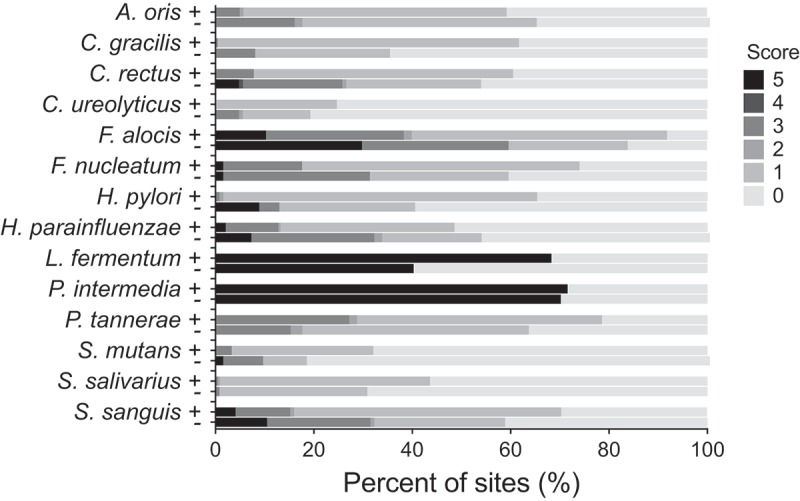
Presence and semi-quantification of *H. pylori* and *C. ureolyticus* and 12 other common dental plaque bacteria in interproximal samples from adult individuals with stomach pain (+) and without (−) of the Karen Hill Tribes of Northern Thailand.

The CKB analyses detected a predominance of some anaerobic species: *F. alocis, F. nucleatum, P. intermedia*, and *P. tannerae* along with the facultative anaerobic species *S. sanguinis* and *H. parainfluenzae*, in the subgingival plaque of individuals of the two groups studied (Figure 1). The two species associated with the gastrointestinal tract *H. pylori* and *C. ureolyticus* were present over the detection level (>10^4^ counts) in 57 and 23% of the samples, respectively (Figure 1). Both bacteria were more prevalent in individuals with stomach pain compared with those without but the difference did not reach statistical significance (Figure 1).

By reducing the high number of bacterial species using factor analyses, the species related to each other in the interproximal plaque were divided into three factors explaining a total of 65% of the variance (Table 2). Factor 1, explaining 43.0% of the variance, forming a cluster consisted of *C. gracilis, C. ureolyticus, H. pylori, L. fermentum, P. tannerae*, and *S. salivarius*, was strongly associated in a multilevel regressions analysis with stomach pain (Table 3, *p* < 0.0001). A second factor which included five anaerobic species *C. rectus, F. alocis, F. nucleatum, P. intermedia*, and *P. tannerae* explained 14.1% of the variance and a Factor 3, including four facultative anaerobic species, *A. oris, H. parainfluenzae, S. mutans*, and *S. sanguinis*, explained 7.6%. Factors 2 and 3 were not significantly associated to stomach pain (Table 3).

**Table 2.** The pattern/structure matrix from factor analyses.^a^

Species	Pattern/Structure coefficients		
Factor analyses (variance)	Factor 1 (43.0 %)	Factor 2 (14.1 %)	Factor 3 (7.6 %)
*A. oris*	0.069	0.414	**0.563**
*C. gracilis*	**0.889**	0.145	0.151
*C. rectus*	0.448	**0.540**	0.294
*C. ureolyticus*	**0.564**	0.040	0.476
*F. alocis*	−0.215	**0.773**	0.170
*F. nucleatum*	0.427	**0.552**	0.274
*H. pylori*	**0.871**	0.148	0.078
*H. parainfluenzae*	−0.107	0.366	**0.778**
*L. fermentum*	**0.909**	0.174	0.057
*P. intermedia*	0.395	**0.708**	0.153
*P. tannerae*	**0.678**	**0.558**	0.037
*S. mutans*	0.338	−0.041	**0.692**
*S. salivarius*	**0.539**	−0.020	0.271
*S. sanguinis*	0.300	0.374	**0.541**

Factor loadings >0.5 are given in bold, indicating a significant contribution to the variance of each factor.

^a^The results are from factor analyses, with Varimax rotation for log counts of identified bacterial species. The three factors illustrated explain 65% of the variance.

**Table 3.** Multilevel regression analyses for factors from factor analyses.

Stomach pain No/Yes	Coefficient	*p*-Value	95% confidence interval	
			Lower	Upper
Factor 1^a^	0.12	0.000*	0.07	0.16
Factor 2^b^	−0.02	0.335	−0.07	0.02
Factor 3^c^	−0.03	0.173	−0.08	0.01

^a^Factor 1 consisting of the following bacteria: *C. gracilis, C. ureolyticus, H. pylori, L. fermentum, P. tannerae, S. salivarius*.

^b^Factor 2 consisting of the following bacteria: *F. alocis, F. nucleatum, C. rectus, P. intermedia, P. tannerae*.

^c^Factor 3 consisting of the following bacteria: *A. oris, H. parainfluenzae, S. mutans, S. sanguinis*.

Logistic regression analyses were performed on subject level variables (Table 4). None of the parameters, age, gender, smoking, betel chewing, or DFT associated significantly with stomach pain. When testing urease activity, no significant association was found for pH, log counts of *H. pylori*, and *C. ureolyticus* (data not shown).

**Table 4.** Logistic regression predicting likelihood of reporting stomach pain.

				95% confidence interval for OR	
Stomach pain No/Yes	*B*	*p*-Value	OR	Lower	Upper
Age	−0.024	0.537	0.976	0.905	1.053
Gender	−0.026	0.959	0.974	0.360	2.635
Smoking	−0.252	0.596	0.778	0.306	1.973
Betel chewing	0.215	0.667	1.240	0.465	3.308
DFT	0.095	0.111	1.100	0.978	1.237
PPD	1.083	0.335	2.954	0.327	26.662
Constant	1.486	0.434	4.419		

## Discussion

The main finding in this study was the high frequency and high counts (>detection level of 10^4^ counts) of the two gastrointestinal pathogens *H. pylori* and *C. ureolyticus* in the dental plaque microbiota of adults that live in remote areas of Northern Thailand and have poor oral hygiene. These results confirm previous findings of a comparatively high prevalence of these two primarily gastrointestinal species in the dental plaque in this population [18,19]. Furthermore, it was found that the presence of these two strongly ureolytic species in the dental biofilm was not associated with stomach pain as single species. However, when clustered together with *P. tannerae, S. salivarius*, and *L. fermentum* in Factor 1 and further analyzed with multilevel regression, they were significantly associated with stomach pain. Stomach pain may thus be reflected in the microbial composition of the dental biofilm microbiota. It is tempting to argue that gastritis and/or peptic ulcer, which are commonly associated with reflux and acidity of the oral cavity [4], can give rise to an environmental change that favors certain bacterial combinations within the oral microbiota. *H. pylori* and *C. ureolyticus* are both strongly ureolytic, which may protect them from pH fluctuations in the oral cavity. Streptococci and lactobacilli are genuinely acid tolerant and are included in the ecological cluster (Factor 1) that came out as significantly associated with stomach pain. Signs of dental wear were noticed, such as attrition and possibly also erosion, which may reflect gastrointestinal reflux disease. Although the association with *H. pylori* and reflux disease was low in a Swedish study [28], we cannot exclude that the presence of *H. pylori* in the oral cavity in adults of this population to some extent may be a result of stomach reflux.

*H. pylori* and *C. ureolyticus* are two bacterial species primarily associated to the gastrointestinal microbiota in humans [12,29]. The occurrence of *H. pylori* in the oral cavity has been shown in numerous studies and the oral cavity has been claimed to function as a reservoir for transmittance to the stomach after *H. pylori* eradication and treatment of peptic ulcers [6]. On the other hand, very little is known about the presence of *C. ureolyticus* and whether it is transient or resident in the oral cavity. In the present study, *H. pylori* and *C. ureolyticus* were frequently detected in high numbers in interproximal plaque samples, which confirms previous studies that showed that these two bacterial species frequently occur in the dental plaque microbiota of this adult population [18,19]. It is therefore suggested that in populations with poor oral hygiene these two species may be regarded as inhabitants of the resident oral microbiota and thereby serve as a reservoir for *H. pylori* and *C. ureolyticus* to be transmitted and reoccur in the gastrointestinal canal. There will be favorable conditions for the two gastrointestinal species, which are microaerophilic (*H. pylori*) or anaerobic (*C. ureolyticus*) to colonize and establish in the dental biofilm in subjects with poor oral hygiene. However, the influence of oral hygiene status with regard to presence of *H. pylori* is controversial and the majority of the studies according to a recent review [6] did not show an association between oral hygiene status and gastric or oral carriage of *H. pylori*, while at least three publications reported that poor oral hygiene was significantly associated with gastric *H. pylori* infection [30–32]. The present study showing a common presence of *H. pylori* and *C. ureolyticus* in interproximal plaque samples supports the view that these two bacteria may easier colonize the oral cavity in a population with poor oral hygiene and thereby constitute a reservoir for further spread to the gastrointestinal tract and increase the risk for stomach pain.

The association of *H. pylori* and *C. ureolyticus* with gingival inflammation and periodontitis is also contradictory [6]. The results of this study showed the predominance of anaerobic bacteria in the interproximal dental plaque and confirmed earlier studies in this population [17–19]. Individuals with poor oral hygiene and long-standing gingivitis have the opportunity to develop a dysbiotic microbiota both supra- and subgingivally, with a lower portion of streptococci, but a higher load of anaerobic bacteria, such as *P. tannerae, F. alocis, F. nucleatum*, and other *Campylobacter* species [18,19]. It is possible that *H. pylori* and *C. ureolyticus* are also favored in the interproximal plaque where no oral hygiene is regularly performed.

The use of the CKB method with its low sensitivity and a detection level of >10^4^ cells [25,27] for evaluation of the subgingival microbiota in this study needs to be commented on. The CKB method was chosen because it is a convenient method in field studies, when sensitivity is a minor problem in samples with high bacterial number due to poor oral hygiene. Another advantage with CKB method is when many samples are taken for testing against a panel of selected bacterial species (in this case 14 species) associated with plaque urease activity and pH [18,20]. The risk of cross-hybridization and lower specificity can be overcome by using high quality and pure specific DNA whole-genomic probes and standards according to a recent methodological report [25]. It is possible that usage of a more sensitive method, like 16S qPCR, would reveal a higher prevalence of these target bacteria including not only *H. pylori* and *C. ureolyticus* but also other urease positive species. Interestingly, another highly urease positive species, *H. parainfluenzae* [23], was commonly present and may contribute to the alkaline environment in the interproximal sites that was found in the resting plaque. This was in particular found in sites of the lower front region, where some extreme values (>8.0) were noticed for some individuals.

The diagnosis ‘stomach pain’ without an objective medical confirmation of gastritis/peptic ulcer is a bias in this study. The subjects were dichotomized into a stomach pain group based on daily pain from the stomach as revealed from interviews but also confirmed by a medical doctor of the team. Nevertheless, the diagnosis of stomach pain involves some uncertainty and the results from the study should be interpreted cautiously. In fact, logistic regression did not reveal any of the subject variables to predict the likelihood for stomach pain. It is not known why this population has such a high rate of stomach pain. It may be speculated that betel chewing plays an important role, but betel chewing was equally common in both groups. Another, putative factor is the diet [2]. The Karen Hill population uses extreme levels of chili, grown and produced in their villages. However, chili has been claimed to be protective against *H. pylori* infection as well as peptic ulcer [33]. The influence of diet on stomach pain and specifically on gastritis and peptic ulcer is still unclear and needs to be studied separately [2].

This study reported a high rate of stomach pain in the Karen Hill tribe population, a population with poor oral hygiene, long-standing gingivitis, and low caries experience. The subjects in the stomach pain group showed a significant correlation with Factor 1, consisting of four oral bacterial species and the two gastrointestinal bacteria, *H. pylori* and *C. ureolyticus*. This study supports the association between stomach pain and a cluster of bacteria including *H. pylori* and *C. ureolyticus*. It indicates for the first time a potential association between *C. ureolyticus* and stomach pain.
